# Screening for cardio-metabolic risk factors in the Romanian cohort of HIV-positive patients

**DOI:** 10.1186/1471-2334-14-S7-P61

**Published:** 2014-10-15

**Authors:** Anca Streinu-Cercel, Oana Săndulescu, Claudiu Mihai Șchiopu, Adrian Streinu-Cercel

**Affiliations:** 1 Carol Davila University of Medicine and Pharmacy, Bucharest, Romania; 2 National Institute for Infectious Diseases "Prof. Dr. Matei Balş", Bucharest, Romania

## Background

HIV is recognized as an independent factor related to cardiovascular (CV) disease [1]. International guidelines recommend evaluation and adjustment of common cardiovascular risk factors for all HIV-positive patients. As antiretroviral (ARV) drugs associate different profiles regarding the CV risk [2], the therapeutic regimens need to be managed on a case-by-case basis [3,4]. The prevalence of CV disease appears to be higher in the HIV population, compared to the general population, and recent studies have demonstrated that the supplementary risk for acute myocardial infarction in HIV-infected patients is 75% [5].

## Methods

We are currently performing a screening study in a cohort of HIV-infected patients to assess cardiovascular and metabolic involvement, through means of: echocardiography, intima-media thickness, Framingham score, serum lipid profile, DXA evaluation, as well as immune and virological evaluation.

## Results

We present the results from a pilot project that included 100 patients from the Romanian HIV cohort. We evaluated 100 patients, with a mean age of 39.8 years old. The male-to-female ratio was 1.7:1. The mean CD4 count was 668 cells/cmm, with 5% of patients presenting CD4 cell counts below 200, 29% between 200-500 and 66% above 500 cells/cmm. The distribution of HIV-RNA was: undetectable (51%), detectable, below 1,000 copies/mL (39%), 1,000-10,000 (4%) and above 10,000 (6%). Upon serum evaluation, 49% of patients had normal cholesterol levels (below 200 mg/dL), 44% had normal triglyceride values (below 150 mg/dL), and 69% had normal glycemic values.

## Conclusion

These preliminary results warrant the continuation of this pilot study, with inclusion of a higher number of patients, in order to reach the project-specified target of enrollment.
